# Identification of cardiomyopathy associated circulating miRNA biomarkers in patients with muscular dystrophy using a complementary cardiovascular magnetic resonance and plasma profiling approach

**DOI:** 10.1186/s12968-016-0244-3

**Published:** 2016-05-06

**Authors:** Svetlana Becker, Anca Florian, Alexandru Patrascu, Sabine Rösch, Johannes Waltenberger, Udo Sechtem, Matthias Schwab, Elke Schaeffeler, Ali Yilmaz

**Affiliations:** Dr. Margarete Fischer-Bosch Institute of Clinical Pharmacology, Stuttgart, Germany; Department of Cardiovascular Medicine, University Hospital Münster, Albert-Schweitzer-Campus 1, building A1, 48149 Münster, Germany; Division of Cardiology, Robert-Bosch-Hospital, Stuttgart, Germany; Department of Clinical Pharmacology, University Hospital Tübingen, Tübingen, Germany; Department of Biochemistry and Pharmacy, University Tübingen, Tübingen, Germany

**Keywords:** miRNA, Cardiomyopathy, Muscular dystrophy, Cardiovascular magnetic resonance, Late gadolinium enhancement

## Abstract

**Background:**

Duchenne and Becker muscular dystrophy (DMD and BMD) are X-chromosomal recessive neuromuscular disorders that are caused by mutations in the dystrophin gene and characterized by cardiac involvement. Circulating microRNAs (miRNAs) have been proposed as diagnostic biomarkers for various cardiovascular diseases. However, circulating miRNAs reflecting the presence and/or disease severity of cardiac involvement in DMD/BMD patients have not been described so far.

**Methods:**

Sixty-three male patients with known MD and 26 age-matched healthy male controls were prospectively enrolled. All MD patients and controls underwent comprehensive cardiovascular magnetic resonance (CMR) studies as well as venous blood sampling on the same day.

**Results:**

An impaired left ventricular (LV) systolic function (defined as LV-EF <55 %) was detected in 29 (46 %) and presence of late gadolinium enhancement (LGE) indicative of myocardial fibrosis in 48 (76 %) MD patients with an exclusively non-ischemic pattern. Whereas no significant differences were observed for the 27 selected circulating miRNAs in MD patients with abnormal CMR findings (comprising structural and/or functional impairments) compared to those with completely normal CMR studies, a significant up-regulation of three miRNAs was observed in LGE-positive MD patients compared to LGE-negative ones: miR-222 (1.8-fold, *p* = 0.035), miR-26a (2.1-fold, *p* = 0.03) and miR-378a-5p (2.4-fold, *p* = 0.026). A signature of these three miRNAs (miR-26a, miR-222 and miR-378a-5p) resulted in an area under the curve (AUC) value of 0.74 for the diagnosis of LGE-positive MD patients. In a multivariable model, three independent predictors for LGE presence were identified comprising not only clinical and laboratory markers (LV-EF: OR 0.47, 95 % CI 0.24-0.89, *p* = 0.021 and elevated hs-Trop: OR 2559, 95 % CI 2.97-22.04*10^5^, *p* = 0.023) but also the circulating miR-222 (OR 938, 95 % CI 938.46, 3.56-24.73*10^4^, *p* = 0.016).

**Conclusions:**

Up-regulation of circulating miRNAs miR-222, miR-26a and miR-378a-5p indicates the presence of myocardial scars in MD patients. Plasma miR-222 appears to be a promising novel biomarker reflecting structural – but not functional – cardiac alterations in MD patients.

**Electronic supplementary material:**

The online version of this article (doi:10.1186/s12968-016-0244-3) contains supplementary material, which is available to authorized users.

## Background

Duchenne and Becker muscular dystrophy (DMD and BMD) are X-chromosomal recessive neuromuscular disorders that are caused by mutations in the dystrophin gene that subsequently lead to either total absence or structural impairment of the dystrophin protein. Since dystrophin is a central protein in the cell membrane of skeletal as well as cardiac muscle cells, MD patients do not only suffer from skeletal muscle weakness and wasting but also from progressive cardiomyopathy [1, 2]. Since therapeutic treatment options for respiratory failure have tremendously improved within the last years, cardiac disease with a characteristic, non-ischemic pattern of left ventricular (LV) myocardial fibrosis leading to non-ischemic dilated cardiomyopathy, heart failure symptoms and ventricular arrhythmias has become a major cause of morbidity and mortality in MD patients [1, 3–5].

Within the last years, cardiovascular magnetic resonance (CMR) has evolved as an excellent tool for the early and sensitive diagnosis of cardiac involvement in MD patients, since this imaging modality does not only allow an accurate functional assessment of the human heart - but also enables non-invasive myocardial fibrosis detection based on techniques such as late gadolinium enhancement (LGE)-imaging or T1-mapping [6–8]. However, previous CMR-based studies in MD patients have also suggested that cardiac serum markers such as troponin or brain-natriuretic peptide (BNP) that are helpful to diagnose and monitor cardiac disease in many ischemic as well as non-ischemic cardiomyopathies, are of limited clinical value in MD patients [9, 10]. Hence, the identification of novel serum biomarkers for diagnosis and monitoring of cardiomyopathy in MD patients (e.g. as a gatekeeper for subsequent CMR studies) is a clinically important, however, still unsolved challenge.

Recent advances in molecular diagnostics have opened novel avenues in defining the molecular basis and underlying pathophysiology of inherited diseases. In particular, microRNAs (miRNAs) have been identified as a novel class of both biomarkers and targets for therapy [11]. miRNAs are small (19–25 nucleotides), non-coding molecules and can modify gene expression by regulating mRNA stability or translation and thereby modify essential cellular functions [12]. Recent studies have shown that miRNAs are not only located inside cells (where they exert their function) but can also be found in different fluids such as blood [13]. Moreover, there is a growing body of evidence that circulating miRNAs can be used as diagnostic as well as prognostic biomarkers for different cardiovascular diseases [14, 15]. However, circulating miRNAs reflecting the presence and/or disease severity of cardiac involvement in DMD/BMD patients have not been described so far.

Hence, the aim of the current study was to identify specific circulating miRNAs in the plasma of DMD/BMD patients that would allow a non-invasive and accurate diagnosis of cardiac disease in these patients.

## Methods

### Study population

As part of an ongoing prospective study, 63 male patients with known MD were prospectively enrolled between 2008 and 2015. A diagnosis of DMD (*N* = 12) or BMD (*N* = 51) has previously been made in specialized neurology centers based on clinical data, skeletal muscle pathology with dystrophin analyses and/or genetic testing [10, 16–18]. The clinical degree of skeletal myopathy was clinically assessed as follows: 0 = no clinical signs of myopathy; 1 = able to walk, unable to run; 2 = unable to walk; 3 = unable to use hands.

In addition, 26 age-matched healthy male controls were enrolled between 2011 and 2014 and represented the control group. All MD patients and controls underwent comprehensive CMR studies as well as venous blood sampling on the same day. The study protocol complies with the Declaration of Helsinki and was approved by the local ethics committee (Ethik-Kommission Landesärztekammer Baden-Württemberg, Stuttgart, Germany). Informed consent was obtained from the patients prior to study inclusion.

### Blood sampling in MD patients and controls

EDTA blood samples were collected on the same day of the CMR study. EDTA plasma was harvested by centrifugation of EDTA Blood Collection Tubes (Sarstedt, Germany) for 10 min at 4000 rpm. Aliquots of plasma supernatant were stored in cryotubes (Sarstedt, Germany) at −20 °C until use. Both in MD patients and controls, laboratory determinations for cardiac biomarkers - high sensitive troponin I (hs-Trop) and brain natriuretic-peptide (NT-proBNP) - were performed using standard methods and considered elevated when serum levels exceeded the upper laboratory reference limit. In addition, creatine kinase (CK) levels were determined.

### miRNA extraction and quantification

RNA was extracted from 400 μl of plasma using mirVana miRNA isolation kit (Life technologies, USA) following the manufacturer’s protocol, eluted in 75 μl elution solution and stored at −80 °C.

miRNA selection for quantification was based on literature data related to either cardiovascular diseases and/or DMD/BMD (Additional file 1: Table S1) were reverse transcribed using TaqMan MicroRNA Reverse Transcription Kit (Life technologies, USA). Individual stem-loop reverse transcription primers included in the predeveloped TaqMan miRNA assay (Life technologies, USA) were pooled at a final dilution of 0.05x for each primer. The final RT reaction volume of 7.5 μl contained 0.15 μl 100 mM dNTP, 1.5 μl multiscribe reverse transcriptase (50 U/μl), 0.75 μl 10 × RT buffer, 0.095 μl RNase inhibitor (20 U/μl), 3 μl primer pool and 2 μl of total RNA. The reaction was performed following conditions of manufacturer.

To improve sensitivity of miRNA quantification, a pre-amplification reaction was performed. TaqMan miRNA assays included in the TaqMan miRNA assay (Life technologies, USA) were pooled at a final dilution of 0.2x for each assay. Pre-amplification reaction was done at 10 μl final volume containing 5 μl TaqMan PreAmp Master Mix (2X), 1.5 μl of assay pool, 2.5 μl of nuclease-free water and 1 μl of cDNA. The pre-amplification PCR was run according to the manufacturer’s protocol, the pre-amplification PCR product was diluted 1:5 with suspension buffer (Teknova AS, Norway) and stored at −20 °C until need.

The miRNA expression levels were quantified by real-time PCR using TaqMan® Universal Master Mix II (no UNG) and TaqMan miRNA assays (Life technologies, USA) on a real-time PCR BioMark system (Fluidigm Corporation, USA) following the manufacturer’s protocol. Relative levels of miRNA expression were calculated by normalization to expression levels of miR-16 and thereafter multiplied by 10^3^ in order to increase readability in the respective tables. The following miRNAs had to be excluded from final analysis due to failing measurements: miR-1, miR-31, miR-34c, miR-95, miR-133a, miR-208a, miR-208b, miR-499a-3p, miR-499a-5p and miR-539.

### CMR data acquisition

ECG-gated CMR studies were performed on a 1.5-T scanner (Aera, Siemens Medical Solutions, Erlangen, Germany) using commercially available cardiac software, electrocardiographic triggering, and cardiac-dedicated surface coils. Cine-imaging was performed using a steady-state-free-precession (SSFP) sequence in three long-axis slices (four-, three- and two-chamber) and a stack of short-axis slices completely covering the LV. LGE-imaging was performed using a T1-weighted inversion recovery gradient-echo sequence 10–15 min after intravenous contrast administration (0.15 mmol/kg Magnevist®) in the same imaging planes as the cine-images.

### CMR data analysis

CMR analysis was performed off-line by two experienced readers blinded to gender and clinical characteristics. Ventricular volumes, ejection fraction and LV mass were derived by contouring endo- and epicardial borders on the short-axis cine images and indexed to body surface area. LGE presence and pattern were first visually assessed on the short-and long-axis images by using the 16-segment AHA model [16]. Second, LGE extent was planimetered on the short-axis contrast images with the use of ImageJ software (National Institutes of Health, Bethesda, Md, USA) and an image intensity level ≥3 SD above the mean of remote myocardium was used to define LGE indicative of damaged myocardium as described previously (National Institutes of Health, Bethesda, Md, USA) and expressed as percentage of total LV mass [19].

An abnormal CMR was defined by at least one of the following findings: i) LV ejection fraction (LV-EF) less than 55 %, ii) RV ejection fraction (RV-EF) less than 45 %, iii) presence of LGE in at least one myocardial segment (AHA segmentation), and was considered as sign of cardiac involvement.

### Statistical analysis

Continuous variables are expressed as mean ± SD. Skewed variables are expressed as median and interquartile range (IQR). Categorical variables are expressed as frequency with percentage. t-Student test was used for comparison of normally distributed variables, while Mann-Whitney U test was used for comparison of non-normally distributed variables. Non-parametric Kruskal–Wallis test with Bonferroni post-hoc correction was used in case of multiple comparisons of non-normally distributed variables. The Chi-square test with Yate’s correction was used to compare non-continuous variables expressed as proportions. Parametric Pearson or non-parametric Spearman correlations were used as corresponded for correlation analysis. In order to find independent predictors for abnormal CMR findings, i.e. LGE presence, a univariable regression analysis was first performed. Second, the parameters with significant p-values were introduced into the multivariable regression analysis. Extremely skewed miRNAs (skewness statistic < −2 or >2) were Log10 transformed before introduced in the regression analysis. Finally, receiver operating characteristic curves (ROC) were analyzed to assess specificity and sensitivity of single plasma miRNAs as well as their combination using multiple logistic regression analysis. Statistical analysis was performed using SPSS software for Windows (version 20, SPSS, Chicago Illinois, US). A *p*-value ≤ 0.05 was considered statistically significant.

## Results

### Patient characteristics

The study group consisted of 63 male MD patients comprising 12 (19 %) patients with DMD and 51 (81 %) with BMD with a median age of 31 ± 15 yrs (Table 1). DMD patients were younger compared to BMD ones (18 ± 5 yrs vs. 33 ± 15 yrs, *p* < 0.0001). The control group comprised 26 healthy male volunteers aged 36 ± 13 yrs (*p* = 0.09). There was no patient with a history of coronary artery disease (CAD) and/or ischemic cardiomyopathy and/or valvular disease. The respective cardiovascular risk profile is illustrated in Table 1. Serum measurements revealed elevated hs-Trop values in 30 (48 %) MD patients (*p* < 0.0001 vs. controls) whereas NT-proBNP levels were increased in 3 (5 %) MD patients only (*p* = 0.55 vs. controls).

**Table 1:** Patient characteristics

	MD	Controls	*p* value
	*N* = 63	*N* = 26	
Male, *n* (%)	63 (100)	26 (100)	1.00
Age, years	31 ± 15	36 ± 13	0.09
BMI, kg/m^2^	24 ± 4	25 ± 3	0.36
Skeletal muscle status 0/1/2/3, *n* (%)	2 (3)/43 (68)/13 (21)/5 (8)	26 (100)/0 (0)/0 (0)/0 (0)	**<0.0001**
Hypertension, *n* (%)	3 (5)	0 (0)	0.55
Diabetes, *n* (%)	0 (0)	0 (0)	1.00
ACE inhibitor, *n* (%)	16 (25)	0 (0)	**0.005**
Beta-blocker, *n* (%)	14 (22)	0 (0)	**0.008**
CK, U/L	1226 (653–2424)	120 (99–169)	**<0.0001**
Elevated hs-Trop, *n* (%)	30 (48)	0 (0)	**<0.0001**
Elevated NT-proBNP, *n* (%)	3 (5)	0 (0)	0.55

Bold text indicates a significant *p*-value of <0.05

### Major CMR findings

**Fig. 1:**
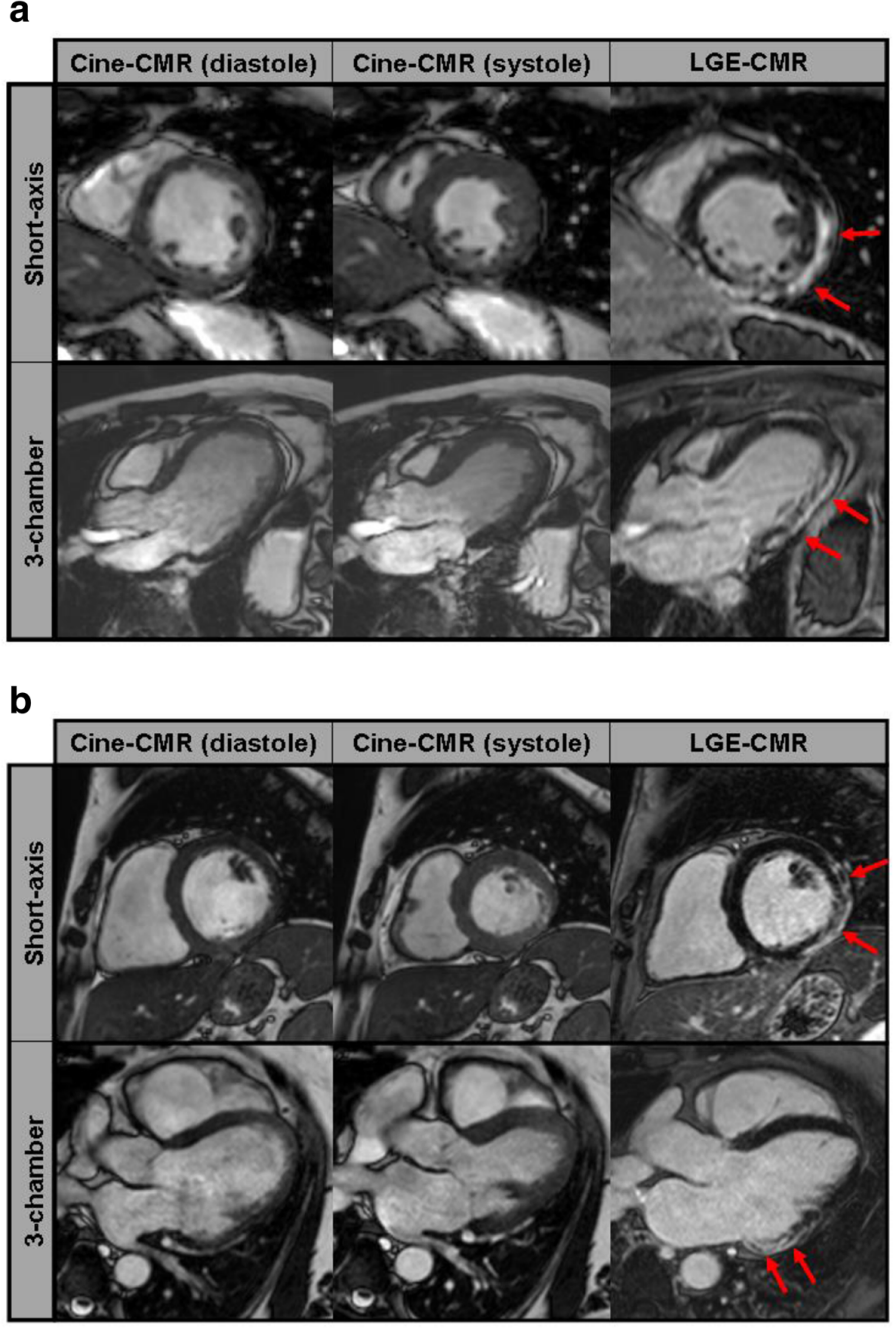
Examples of cine- and late gadolinium enhancement (LGE)-CMR images of a patient with Duchenne (**a**) and Becker muscular dystrophy (**b**) in short-axis views (upper panels) and 3-chamber views (lower panels). In both, the same non-ischemic, predominantly subepicardial LGE pattern (arrows) indicating myocardial fibrosis can be seen.

The detailed results of the CMR studies performed in MD patients and controls are given in Table 2. An impaired LV systolic function (defined as LV-EF <55 %) was detected in 29 (46 %) MD patients (*p* < 0.0001 vs. controls) and a reduced RV systolic function (defined as RV-EF <45 %) in 10 (16 %) MD patients (*p* = 0.031 vs. controls). Presence of LGE indicative of myocardial fibrosis was detected in 48 (76 %) MD patients (*p* < 0.0001 vs. controls) with a median LGE extent of 10 % (IQR 5 %–24 %) of LV mass and an exclusively non-ischemic pattern (Fig. 1a-b). There was a strong negative correlation between LGE extent and LV-EF (Spearman’s rho = -0.753, *p* < 0.0001). Altogether, any pathological CMR finding was observed in 49 (78 %) MD patients whereas all subjects in the control group demonstrated normal CMR findings (*p* < 0.0001).

**Table 2:** Overview CMR results

	MD	Controls	*p* value
	*N* = 63	*N* = 26	
LV-EDVi, ml/m^2^	85 ± 30	86 ± 16	0.80
LV-ESVi, ml/m^2^	41 ± 21	31 ± 9	**0.003**
LV-massi, g/m^2^	60 ± 16	58 ± 10	0.69
LV-EF, %	53 ± 12	64 ± 6	**<0.0001**
RV-EF, %	53 ± 9	55 ± 8	0.47
LV-EF <55 %, *n* (%)	29 (46)	0 (0)	**<0.0001**
RV-EF <45 %, *n* (%)	10 (16)	0 (0)	**0.031**
LGE presence, *n* (%)	48 (76)	0 (0)	**<0.0001**
LGE extent, %	7 (1-17)	-	-
Abnormal CMR, *n* (%)	49 (78)	0 (0)	**<0.0001**

Bold text indicates a significant *p*-value of <0.05

No significant differences were seen between DMD and BMD neither in frequencies of LV and/or RV systolic impairment (42 % vs. 47 %, *p* = 1.00 for LV; 25 % vs. 14 %, *p* = 0.39 for RV) nor in LGE presence (92 % vs. 73 %, *p* = 0.26) or LGE extent. Consequently, there was also no significant difference in the prevalence of an abnormal CMR study between these groups (92 % vs. 75 %, *p* = 0.27).

### miRNA findings in MD patients vs. controls

Plasma miRNA results are shown in Table 3. A significant up-regulation in MD patients compared to controls was found for seven out of 17 plasma miRNAs included in the analysis: miR-206 (91-fold increase, *p* < 0.0001), miR-20b (1.5-fold increase, *p* = 0.048), miR-222 (24-fold, *p* < 0.0001), miR-26a (6-fold, *p* < 0.0001), miR-342 (33-fold, *p* < 0.0001), miR-378a-3p (minimum 3000-fold; almost undetectable in controls, *p* < 0.0001), miR-378a-5p (48-fold, *p* < 0.0001). Additionally, a significant down-regulation was found for the three miRNAs: miR-221, miR-29a and miR-29c, all almost undetectable in the plasma of MD patients.

**Table 3:** Plasma miRNA results in MD patients vs. controls

miRNA plasma levels^a^ (/10^3^)	MD	Controls	*p* value
	*N* = 63	*N* = 26	
-206	17.20 (4.29–63.96)	0.19 (0.12–0.64)	**<0.0001**
-144-5p	9.85 (0.00–169.32)	13.16 (6.53–19.63)	0.81
-146b	62.55 (0.00–176.97)	16.64 (6.86–33.02)	0.16
-15b	0.00 (0.00–19.61)	2.46 (0.81–3.93)	0.64
-195	9.01 (0.52–21.73)	13.63 (10.46–16.81)	0.053
-20b	61.74 (21.70–85.10)	35.95 (27.39–51.02)	**0.048**
-21	30.61 (0.00–110.97)	9.58 (5.86–18.33)	0.48
-221	0.00 (0.00–20.44)	10.61 (7.73–17.12)	**0.001**
-222	1923.17 (881.56–5111.93)	79.22 (43.34–169.52)	**<0.0001**
-26a	293.34 (121.00–554.69)	49.40 (26.58–63.10)	**<0.0001**
-29a	0.00 (0.00–0.00)	0.07 (0.00–1.11)	**0.002**
-29c	0.00 (0.00–1.43)	0.56 (0.27–2.72)	**0.001**
-342	2427.02 (1198.59–4185.82)	73.89 (45.30–171.19)	**<0.0001**
-378a-3p	30 (5.07–268.79)	0.00 (0.00–0.23)	**<0.0001**
-378a-5p	27.57 (8.84–81.04)	0.58 (0.30–1.79)	**<0.0001**
-451	184.13 (73.28–343.07)	144.45 (107.74–169.24)	0.19
-93	26.82 (0.00–66.29)	35.37 (25.60–49.70)	0.24

(^a^) – Each miRNA plasma level was normalized to miR-16 plasma levels and thereafter multiplied by 10^3^ in order to increase readability

Bold text indicates a significant *p*-value of <0.05

Among MD patients, none of the miRNAs differed significantly in patients with elevated vs. normal hs-Trop and NT-proBNP, respectively. Moreover, we did not detect any significant differences in miRNA expression in MD patients with mild myopathy (clinical degree 0-1 as defined in our Methods section) compared to those with advanced/severe myopathy (clinical degree of 2–3) (Additional file 2: Table S2).

When we assessed the relationship between plasma miRNAs and age, significant but moderate correlations were found only for two of them: miR-26a (Spearman’s rho = +0.312, *p* = 0.013) and miR-378a-3p (Spearman’s rho = −0.299, *p* = 0.017) in the MD group. In contrast, no significant relationship was found in controls regarding plasma miRNA levels and age.

### Assessment of CMR and miRNA findings considering all CMR results

When MD patients with any abnormal CMR findings (*n* = 49; 78 %) were compared to those without (*n* = 14; 22 %), there was no significant difference regarding age or MD type (Tables 4 and 5). An elevated hs-Trop plasma level was more frequently observed in patients with abnormal CMR findings compared to those without. However, only 57 % (*n* = 28) of those patients with pathological CMR findings also demonstrated elevation of hs-Trop. Moreover, there were no significant differences in NT-proBNP levels between MD patients with abnormal CMR findings and those without.

**Table 4:** Patient characteristics and cardiac findings according to CMR results

	MD	Normal CMR	Abnormal CMR	*p* value
	*N* = 63	*N* = 14	*N* = 49	
Male, *n* (%)	63 (100)	14 (100)	49 (100)	1.00
Age, years	31 ± 15	25 ± 18	32 ± 14	0.11
BMD, *n* (%)	51 (81)	13 (93)	38 (78)	0.27
BMI, kg/m^2^	24 ± 4	23 ± 6	24 ± 4	0.75
Skeletal muscle status 0/1/2/3, *n* (%)	2 (3)/43 (68)/13 (21)/5 (8)	2 (14)/10 (71)/1 (7)/1 (7)	33 (67)/12 (25)/4 (8)/0 (0)	0.07
ACE inhibitor, *n* (%)	16 (25)	1 (7)	15 (31)	0.09
Beta-blocker, *n* (%)	14 (22)	1 (7)	13 (27)	0.16
CK, U/L	1226 (653-2424)	764 (596-2411)	1344 (783-2463)	0.24
Elevated hs-Trop, *n* (%)^a^	30 (48)	2 (14)	28 (57)	**0.006**
Elevated NT-proBNP, *n* (%)^b^	3 (5)	0 (0)	3 (6)	1.00

^a^ > 14 pg/mL; ^b^ > 450 pg/mL

Bold text indicates a significant *p*-value of <0.05

**Table 5:** Cardiac findings according to CMR results

	MD	Normal CMR	Abnormal CMR	*p* value
	*N* = 63	*N* = 14	*N* = 49	
LV-EDVi, ml/m^2^	85 ± 30	72 ± 19	88 ± 32	0.08
LV-ESVi, ml/m^2^	41 ± 21	24 ± 7	46 ± 21	**<0.0001**
LV-mass, g/m^2^	60 ± 16	52 ± 11	62 ± 17	**0.037**
LV-EF, %	53 ± 12	67 ± 6	50 ± 11	**<0.0001**
RV-EF, %	53 ± 9	58 ± 7	52 ± 9	**0.023**
LV-EF <55 %, *n* (%)	29 (46)	0 (0)	29 (59)	NA
RV-EF <45 %, *n* (%)	10 (16)	0 (0)	10 (20)	NA
LGE presence, *n* (%)	48 (76)	0 (0)	48 (98)	NA
LGE extent, %	7 (1–17)	-	10 (4–24)	NA

Bold text indicates a significant *p*-value of <0.05

As shown in Table 6, there were no significant differences in any of the measured plasma miRNAs in MD patients with any abnormal CMR finding (comprising structural and/or functional impairments) compared to those with completely normal CMR studies.

**Table 6:** Plasma miRNA results in MD patients with normal vs. pathological CMR results

miRNA plasma levels^a^ (/10^3^)	Normal CMR	Abnormal CMR	*p* value
	*N* = 14	*N* = 49	
-206	14.36 (3.07–49.46)	21.76 (4.82–72.71)	0.68
-144-5p	8.94 (0.00–163.41)	9.85 (0.00–171.21)	0.97
-146b	84.15 (40.30–169.65)	38.75 (0.00–177.31)	0.25
-15b	7.44 (0.00–21.87)	0.00 (0.00–18.78)	0.43
-195	9.76 (1.53–19.84)	8.31 (0.57–21.25)	0.86
-20b	49.38 (29.88–79.03)	65.38 (19.43–86.37)	0.66
-21	71.91 (2.89–107.05)	11.48 (0.00–113.69)	0.61
-221	0.00 (0.00–35.26)	0.00 (0.00–11.27)	0.87
-222	1367.66 (689.45–1764.90)	2230.30 (964.52–5884.91)	0.09
-26a	173.94 (47.45–324.42)	326.81 (126.07–602.29)	0.63
-29a	0.00 (0.00–0.00)	0.00 (0.00–0.00)	0.94
-29c	0.00 (0.00–2.24)	0.00 (0.00–0.56)	0.93
-342	1987.68 (808.31–2723.93)	2639.34 (1333.85–5106.77)	0.16
-378a-3p	14.15 (0.00–273.74)	39.74 (5.89–290.67)	0.39
-378a-5p	17.32 (2.66–40.41)	39.29 (13.24–101.0)	0.06
-451	104.66 (57.69–312.21)	206.41 (110.79–338.52)	0.27
-93	24.46 (0.38–55.52)	26.95 (0.00–69.92)	0.93

(^a^) – Each miRNA plasma level was normalized to miR-16 plasma levels and thereafter multiplied by 10^3^ in order to increase readability

Bold text indicates a significant *p*-value of <0.05

### Assessment of CMR and miRNA findings according to “functional” CMR results

Fifty-one percent (*n* = 32) of the MD patients showed a reduced LV and/or RV systolic function in the CMR study as measured by the respective EF. In this subgroup of MD patients with impaired systolic function, both LGE prevalence and extent were significantly increased compared to those MD patients with normal biventricular systolic function (*n* = 31; 49 %) as depicted in Tables 7 and 8.

**Table 7:** Patient characteristics focusing on functional CMR results

	MD	Normal LV and RV function	Impaired LV and/or RV function	*p* value
	*N* = 63	*N* = 31	*N* = 32	
Male, *n* (%)	63 (100)	31 (100)	32 (100)	1.00
Age, years	31 ± 15	27 ± 15	34 ± 15	0.11
BMD, *n* (%)	51 (81)	26 (84)	25 (78)	0.75
BMI, kg/m^2^	24 ± 4	24 ± 5	24 ± 3	0.72
Skeletal muscle status 0/1/2/3, *n* (%)	2 (3)/43 (68)/13 (21)/5 (8)	2 (7)/23 (74)/5 (16)/1 (3)	20 (63)/8 (25)/4 (13)/0 (0)	0.20
ACE inhibitor, *n* (%)	16 (25)	2 (7)	14 (44)	**0.001**
Beta-blocker, *n* (%)	14 (22)	4 (13)	10 (31)	0.13
CK, U/L	1226 (653–2424)	1250 (652–3043)	1221 (727–2291)	0.86
Elevated hs-Trop, *n* (%)^a^	30 (48)	8 (26)	22 (67)	**0.001**
Elevated NT-proBNP, *n* (%)^b^	3 (5)	0 (0)	3 (9)	0.24

^a^ > 14 pg/mL; ^b^ > 450 pg/mL

Bold text indicates a significant *p*-value of <0.05

**Table 8:** Cardiac findings focusing on functional CMR results

	MD	Normal LV and RV function	Impaired LV and/or RV function	*p* value
	*N* = 63	*N* = 31	*N* = 32	
LV-EDVi, ml/m^2^	85 ± 30	73 ± 18	96 ± 35	**0.003**
LV-ESVi, ml/m^2^	41 ± 21	27 ± 9	54 ± 21	**<0.0001**
LV-mass, g/m^2^	60 ± 16	53 ± 11	66 ± 18	**0.001**
LV-EF, %	53 ± 12	63 ± 6	44 ± 9	NA
RV-EF, %	53 ± 9	58 ± 7	49 ± 9	NA
LV-EF <55 %, *n* (%)	29 (46)	0 (0)	29 (91)	NA
RV-EF <45 %, *n* (%)	10 (16)	0 (0)	10 (31)	NA
LGE presence, *n* (%)	48 (76)	17 (55)	31 (97)	**<0.0001**
LGE extent, %	7 (1–17)	1 (0–6)	16 (8–32)	**<0.0001**
Abnormal CMR, *n* (%)	49 (78)	17 (55)	32 (100)	NA

Bold text indicates a significant *p*-value of <0.05

However, there were again no significant differences in any of the measured plasma miRNAs in MD patients with an impaired systolic function compared to those without (Table 9). The only significant correlation between a miRNA and LV-EF was a moderate negative one for miR-29c (Spearman’s rho = −0.323, *p* = 0.010).

**Table 9:** Plasma miRNA results in MD patients with normal vs. impaired systolic function

miRNA plasma levels^a^ (/10^3^)	Normal LV and RV function	Impaired LV and/or RV function	*p* value
	*N* = 31	*N* = 32	
-206	31.77 (4.83–79.62)	11.27(3.52–50.27)	0.58
-144-5p	33.14 (0.00–525.08)	0.00 (0.00–98.72)	0.29
-146b	74.79 (0.00–173.85)	29.92 (0.00–177.79)	0.60
-15b	0.00 (0.00–18.15)	0.18 (0.00–21.81)	0.77
-195	9.01 (0.35–18.62)	8.93 (1.90–22.0)	0.66
-20b	53.99 (25.84–83.60)	65.45 (19.35–87.12)	0.66
-21	42.09 (0.00–102.71)	13.65 (0.00–150.01)	0.41
-221	0.00 (0.00–18.27)	0.00 (0.00–18.55)	0.36
-222	1801.09 (1215.81–3491.60)	2006.29 (638.43–6059.36)	0.79
-26a	283.14 (110.62–512.14)	362.31 (121.25–622.72)	0.36
-29a	0.00 (0.00–0.00)	0.00 (0.00–0.05)	0.39
-29c	0.00 (0.00–0.47)	0.00 (0.00–2.16)	0.27
-342	2249.28 (1294.10–3353.27)	2570.15 (888.62–5565.11)	0.54
-378a-3p	28.84 (6.04–155.98)	32.92 (1.76–301.61)	0.98
-378a-5p	27.57 (12.50–56.49)	26.02 (7.00–84.56)	0.78
−451	184.13 (100.07–349.44)	171.93 (22.68–307.38)	0.77
−93	26.19 (0.00–70.81)	27.17 (0.00–53.75)	0.81

(^a^) – Each miRNA plasma level was normalized to miR-16 plasma levels and thereafter multiplied by 10^3^ in order to increase readability

Bold text indicates a significant *p*-value of <0.05

### Assessment of CMR and miRNA findings according to “structural” CMR results

In the next step, MD patients with presence of structural abnormalities - defined as presence of LGE (*n* = 48; 76 %) - were compared to those without any LGE (*n* = 15; 24 %). LGE-positive MD patients showed more frequently a reduced LV-EF (28 (58 %) vs. 1 (7 %); *p* = 0.001) and an elevated hs-Trop level (28 (58 %) vs. 2 (13 %); *p* = 0.003) compared to LGE-negative ones (Tables 10 and 11).

**Table 10:** Patient characteristics focusing on structural CMR results

	MD	LGE-negative	LGE-positive	*p* value
	*N* = 63	*N* = 15	*N* = 48	
Male, *n* (%)	63 (100)	15 (100)	48 (100)	1.00
Age, years	31 ± 15	25 ± 17	32 ± 14	0.13
BMD, *n* (%)	51 (81)	14 (93)	37 (77)	0.26
BMI, kg/m^2^	24 ± 4	23 ± 5	24 ± 4	0.38
Skeletal muscle status 0/1/2/3, *n* (%)	2 (3)/43 (68)/13 (21)/5 (8)	2 (13)/11 (73)/1 (7)/1 (7)	32 (67)/12 (25)/4 (8)/0 (0)	0.06
ACE inhibitor, *n* (%)	16 (25)	1 (7)	15 (31)	0.09
Beta-blocker, *n* (%)	14 (22)	1 (7)	13 (27)	0.16
CK, U/L	1226 (653–2424)	773 (607–2187)	1358 (797-2481)	0.18
Elevated hs-trop, *n* (%)^a^	30 (48)	2 (13)	28 (58)	**0.003**
Elevated NT-proBNP, *n* (%)^b^	3 (5)	0 (0)	3 (6)	1.00

^a^ > 14 pg/mL; ^b^ > 450 pg/mL

Bold text indicates a significant *p*-value of <0.05

**Table 11:** Cardiac findings focusing on structural CMR results

	MD	LGE-negative	LGE-positive	*p* value
	*N* = 63	*N* = 15	*N* = 48	
LV-EDVi, ml/m^2^	85 ± 30	72 ± 18	89 ± 32	0.06
LV-ESVi, ml/m^2^	41 ± 21	25 ± 7	46 ± 22	**<0.0001**
LV-mass, g/m^2^	60 ± 16	51 ± 11	62 ± 17	**0.018**
LV-EF, %	53 ± 12	66 ± 6	50 ± 11	**<0.0001**
RV-EF, %	53 ± 9	56 ± 10	52 ± 9	0.15
LV-EF <55 %, *n* (%)	29 (46)	1 (7)	28 (58)	**0.001**
RV-EF <45 %, *n* (%)	10 (16)	1 (7)	9 (19)	0.43
LGE extent, %	7 (1–17)	-	10 (5–24)	NA
Abnormal CMR, *n* (%)	49 (78)	1 (7)	48 (100)	NA

Bold text indicates a significant *p*-value of <0.05

As illustrated in Table 12, a significant up-regulation of three miRNAs was observed in LGE-positive MD patients compared to LGE-negative ones: miR-222 (1.8-fold, *p* = 0.035), miR-26a (2.1-fold, *p* = 0.03) and miR-378a-5p (2.4-fold, *p* = 0.026). There was no miRNA that was significantly down-regulated in the LGE-positive group compared to the LGE-negative one.

**Table 12:** Plasma miRNA results in MD patients with vs. without presence of LGE

miRNA plasma levels^a^ (/10^3^)	LGE-negative	LGE-positive	*p* value
	*N* = 15	*N* = 48	
-206	13.18 (3.69–43.72)	22.85 (4.56–74.83)	0.54
-144-5p	15.70 (0.00–159.39)	4.93 (0.00–191.57)	0.95
-146b	81.86 (32.74–162.66)	44.23 (0.00–177.79)	0.32
-15b	7.42 (0.00–21.10)	0.00 (0.00–19.01)	0.41
-195	10.50 (2.70–17.48)	8.19 (0.54–21.93)	0.75
-20b	46.56 (31.69–74.64)	65.45 (19.35–86.58)	0.58
-21	67.48 (5.74–106.23)	11.82 (0.00–115.78)	0.65
-221	0.00 (0.00–32.92)	0.00 (0.00–10.03)	0.66
-222	1288.92 (355.69–1728.72)	2303.59 (1102.54–6059.36)	**0.035**
-26a	165.39 (36.75–291.31)	345.01 (139.78–616.20)	**0.030**
-29a	0.00 (0.00–.09)	0.00 (0.00–0.00)	0.61
-29c	0.00 (0.00–2.14)	0.00 (0.00–.65)	0.89
-342	1906.24 (491.08–2603.63)	2668.49 (1512.77–5205.06)	0.07
-378a-3p	13.18 (0.00–155.98)	40.31 (8.05–301.61)	0.26
-378a-5p	16.99 (1.04–36.13)	41.23 (14.62–102.03)	**0.026**
-451	106.88 (64.56–273.40)	207.05 (100.16–340.80)	0.29
-93	23.85 (0.77–52.24)	27.17 (0.00–70.37)	0.89

(^a^) – Each miRNA plasma level was normalized to miR-16 plasma levels and thereafter multiplied by 10^3^ in order to increase readability

Bold text indicates a significant *p*-value of <0.05

**Fig. 2:**
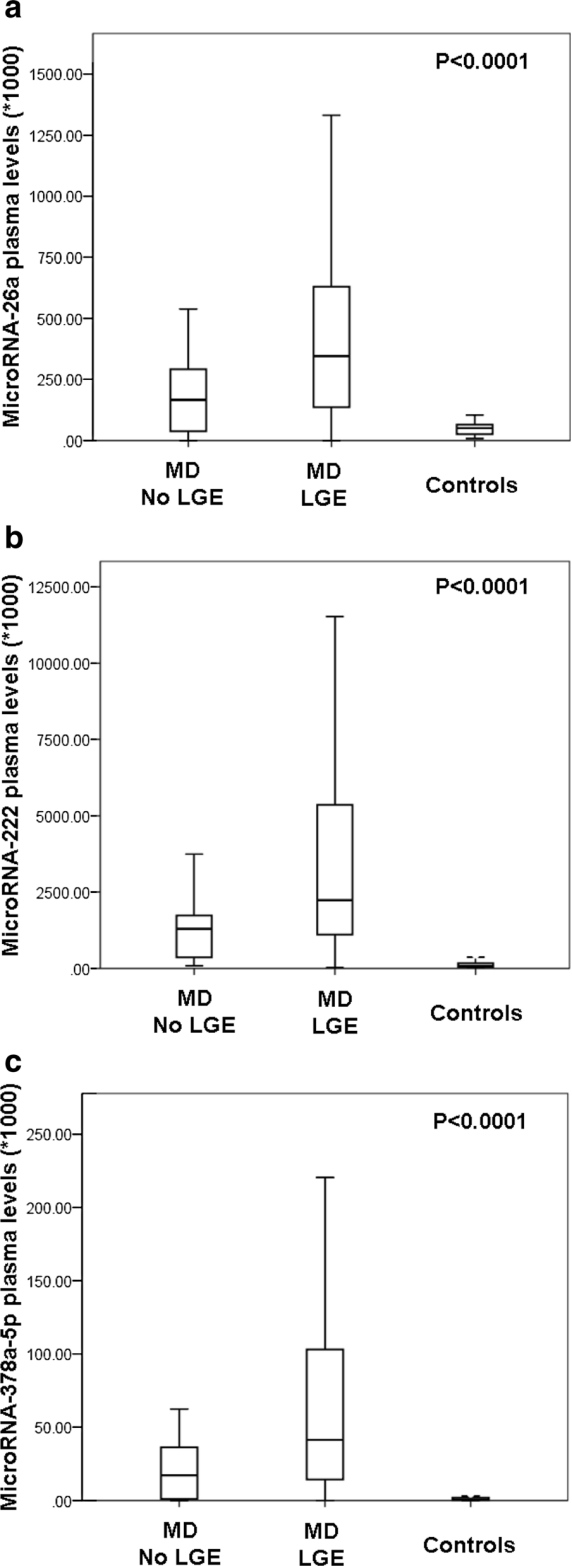
Expression of circulating miRNA-26a (**a**), miRNA-222 (**b**) and miR-378a-5p (**c**) in plasma of LGEnegative (*N* = 15) and LGE-positive (*N* = 48) muscular dystrophy patients as well as controls (*N* = 26). Each miRNA plasma level was normalized to miR-16 plasma levels and thereafter multiplied by 103 in order to increase readability. LGE: late gadolinium enhancement

When the miRNA results of LGE-positive and LGE-negative patients were compared to controls, there were significant differences when all three groups were considered (Fig. 2a-c). However, when post hoc analysis for multiple group comparisons was performed, differences between MD patients with and without LGE were only borderline significant for any of the miRNAs – as expected in case of multiple testing in a small-sized study group. While in this post hoc comparison circulating miR-222 and miR-378a-5p levels were significantly higher both in LGE-positive and LGE-negative patients compared to controls (*p* = 0.003 and *p* < 0.0001 for miR-222; *p* = 0.017 and *p* < 0.0001 for miR-378a-5p), a significant difference was noted only between LGE-positive patients and controls for miR-26a (*p* < 0.0001) – but not for LGE-negative ones vs. controls.

### Assessment of possible predictors of LGE presence

In order to further characterize the association between myocardial scarring as detected by LGE-CMR and the three up-regulated plasma miRNAs, we first performed univariable logistic regression analyses for a series of potential predictors of LGE occurrence - including miR-222, miR-26a and miR-378a-5p (Table 13). In this analysis, a significant association with LGE presence was found for the following parameters: a) CMR parameters such as LV-EF, LV-ESVi and LV-mass, b) the serum marker elevated hs-Trop and c) the miRNAs miR-222 and miR-26a.

**Table 13:** Univariable analysis regarding predictors for LGE presence

Variable (*N* = 63)	OR (95 % CI)	*p* value
Age, years	1.03 (0.99–1.08)	0.13
BMD, *n* (%)	4.16 (0.49–35.28)	0.19
Skeletal muscle status 0/1/2/3, n (%)	2.83 (0.84–9.50)	0.09
Log_10_ CK, U/L	1.69 (0.39–7.36)	0.48
LV-EDVi, ml/m^2^	1.02 (0.99–1.05)	0.06
LV-ESVi, ml/m^2^	1.11 (1.04–1.19)	**0.003**
LV-mass, g/m^2^	1.06 (1.01–1.11)	**0.024**
LV-EF, %	0.81 (0.71–0.91)	**0.001**
Elevated hs-Trop, *n* (%)^a^	9.1 (1.85–44.87)	**0.007**
miR-222 (/10^3^)^c,d^	2.51 (1.01–6.21)	**0.047**
miR-378a-5p (/10^3^)^c,d^	1.00 (0.99–1.01)	0.86
miR-26a (/10^3^)^c^	12.86 (1.07–154.34)	**0.044**

^a^ > 14 pg/mL

(^b^) – Each miRNA plasma level was normalized to miR-16 plasma levels and thereafter multiplied by 10^3^ in order to increase readability

(^c^) - Log_10_ transformed before introduced in the regression analysis

Bold text indicates a significant *p*-value of <0.05

In the next step, we performed a multivariable regression analysis focusing on four variables of most interest among those being statistically significant variables from the aforementioned univariable analysis (LV-EF, elevated hs-Trop, miR-222 and miR-26a). In this multivariable model, three independent predictors for LGE presence were found: a) LV-EF (OR 0.47, 95 % CI 0.24-0.89, *p* = 0.021), b) an elevated hs-Trop (OR 2559, 95 % CI 2.97-22.04*10^5^, *p* = 0.023) and c) serum miR-222 levels (OR 938, 95 % CI 3.56-24.73*10^4^, *p* = 0.016).

### Plasma miRNAs for the identification of MD patients with LGE presence

**Fig. 3:**
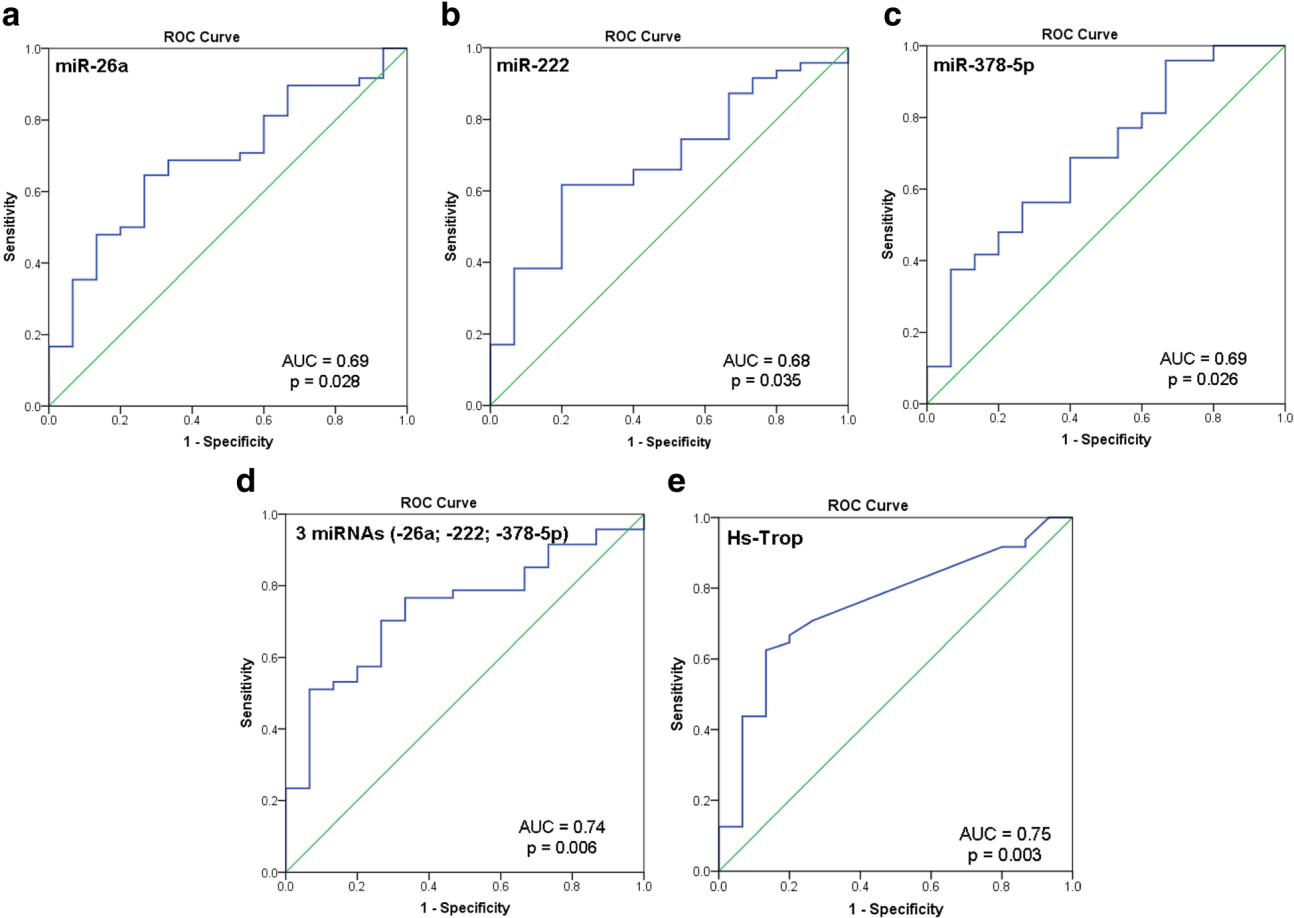
Receiver operating characteristic (ROC) analysis of individual (**a**-**c**) and combined (**d**) plasma miRNA-26a, miRNA-222 and miR-378a-5p to discriminate LGE-negative (*N* = 15) from LGE-positive (*N* = 48) muscular dystrophy patients. Each miRNA plasma level was normalized to miR-16 plasma levels and thereafter multiplied by 10^3^ in order to increase readability. **e** ROC analysis for the conventional serum marker high-sensitive troponin (hs-Trop). LGE, late gadolinium enhancement. AUC, area under the curve

As shown in Fig. 3a-c, the individual receiver operating characteristics curves (ROC) for the three significantly up-regulated miRNAs in MD patients with vs. without LGE revealed areas under the curve (AUC) close to 0.70. Sensitivities, specificities and overall accuracies for these miRNAs were as follows: a) miR-26a: 65, 73 and 67 % (cut-off value of 241.54*10-3), b) miR-222: 62, 80 and 65 % (cut-off value of 1833.18*10-3) and c) miR-378a-5p: 69, 60 and 67 % (cut-off value of 18.84*10-3).

Furthermore, by combining these three miRNAs (miR-26a, miR-222 and miR-378a-5p, Fig. 3d) as potential diagnostic signature for cardiomyopathy in MD patients, an improved AUC value of 0.74 was reached with a sensitivity of 70 %, a specificity of 73 % and an overall accuracy of 70 %. In comparison, ROC analysis for the conventional serum marker hs-Trop regarding the identification of LGE-positive MD patients revealed an AUC value of 0.75 with a sensitivity of 63 %, a specificity of 87 % and an overall accuracy of 65 % (Fig. 3e). Moreover, there was no significant correlation between hs-Trop and each of the three miRNAs (miR-26a, miR-222 and miR-378a-5p).

## Discussion

To the best of our knowledge, this is the first study that assessed the diagnostic value of circulating miRNAs for the detection and prediction of functional as well as structural cardiac impairments (that were assessed by comprehensive CMR studies) in MD patients. The present results indicate that some circulating miRNAs are differently expressed in the plasma of MD patients compared to healthy volunteers. However, for the first time we also identified three circulating miRNAs (miR-26a, miR-222 and miR-378a-5p) that were significantly up-regulated in MD patients with proof of myocardial scarring (based on LGE-CMR) compared to those without – independent of the patients’ age and skeletal muscle status. Interestingly, the association between the plasma profile of these three miRNAs and the presence of cardiac disease was only observed in case of structural changes (proof of myocardial scarring) – but not in case of exclusive functional abnormalities.

### Pathophysiology of cardiac involvement in MD

As outlined previously, the detailed molecular pathomechanism leading to cardiac disease in MD patients is still to be elucidated, although the underlying genetic dystrophin defect can be identified easily by appropriate mutation screening [1]. Based on preclinical studies in dystrophin deficient animal models, early alterations in cardiomyocyte metabolism and signal transduction were suggested [20]. In addition, excessive intracellular calcium signalling and reactive oxygen species (ROS) generation with breakdown of the mitochondrial membrane potential were described and may constitute the link between the initial sarcolemmal injury due to dystrophin deficiency and mitochondrial dysfunctions. Of note, De Arcangelis et al. showed in an animal model that miRNAs are involved in the regulation of the dystrophin-glycoprotein complex components in dystrophic muscles of the mdx mice. Especially, miR-222 expression was increased in dystrophic muscle and led to a decrease of the b1-syntrophin expression [21]. Taken together, the fragility of the cell membrane that is caused by deficient sarcolemmal dystrophin may predispose not only skeletal muscle cells but also cardiomyocytes to metabolic dysfunctions, which in turn may be enhanced by mechanical stress.

In recent years, CMR imaging has gained wide acceptance for non-invasive evaluation of ischaemic as well as non-ischaemic cardiomyopathies since multi-parametric CMR does not only allow the assessment of functional parameters (such as systolic function) but also enables a non-invasive assessment of myocardial scarring based on LGE-imaging or T1-mapping [22]. Within the last years, the distribution pattern of myocardial scarring in MD patients (as well as female MD carriers) was assessed based on such comprehensive CMR studies and it could be consistently shown that the posterolateral LV wall segments represent the first and most extensive sites of myocardial fibrosis in MD patients – as well as their female MD carrier relatives [17, 23–25] (Fig. 1). Moreover, we could recently show that a “transmural” pattern of myocardial fibrosis independently predicts the occurrence of adverse cardiac events in DMD/BMD patients [23].

### Conventional serum markers of cardiac disease in MD patients

Today, measurement of serum BNP levels is routinely performed in patients presenting with heart failure symptoms and/or suspected cardiomyopathy (e.g. dilated cardiomyopathy) since normal BNP values allow to elegantly rule out congestive heart failure whereas elevated BNP values (in patients with normal renal function) point to an underlying cardiac disease and necessitate further cardiac examinations such as echocardiography and/or CMR [9]. Interestingly, in the present study (comprising 63 MD patients), elevated Nt-proBNP levels were measured in three (5 %) MD patients only – although any abnormal cardiac findings were detected in 49 (78 %) and a reduced LV-EF was observed in 29 (46 %) of them by CMR. Hence, measurement of serum BNP is not very sensitive for diagnosing or ruling out cardiac disease in MD patients. As discussed previously, one explanation for this observation may be that serum BNP levels are supposed to reflect rapid changes of pressure gradients in the atria and ventricles and one may argue that the underlying pathophysiology in MD patients is mainly driven by continuous myocardial cell death (due to dystrophin absence or fragility) resulting secondarily in a continuous and adapted ventricular enlargement with progressive decrease of systolic function, however, without quick changes in atrial/ventricular filling pressures [10, 26, 27].

Furthermore, measurement of serum troponin is routinely performed nowadays not only in patients with acute chest pain syndromes but also in those with (suspected) non-ischemic cardiomyopathy for further detailed classification and risk stratification of such patients [28, 29]. In particular, an elevated level of high-sensitive troponin is believed to be an accurate serum marker of myocardial cell death in various acute settings. In the present study, an elevated serum level of hs-Trop was detected in 30 (48 %) MD patients only - although (as aforementioned) any abnormal cardiac findings were detected in 49 (78 %) and proof of myocardial scarring/damage in 48 (76 %) of the study MD patients by CMR. Hence, measurement of serum hs-Trop in MD patients does not allow to detect all of those MD patients who have at least some structural cardiac abnormalities - as can be depicted by LGE-CMR. Moreover, even hs-Trop is not 100 % specific for cardiac disease and may also originate from cell death of skeletal muscle myocytes – particularly in patients with neuromuscular disorders and advanced skeletal myopathy. Taken together, a normal hs-Trop level in a MD patient does not always rule out cardiac disease and in contrast, an elevated hs-Trop level does not necessarily indicate the presence of cardiac abnormalities. Therefore, novel (more sensitive as well as more specific) markers are wanted for early and accurate diagnosis of cardiac involvement in MD patients.

### Circulating miRNAs as novel biomarkers of cardiac disease in MD patients

Recent advances in molecular diagnostics have shown that circulating miRNAs can be used as diagnostic as well as prognostic biomarkers for different cardiovascular diseases [14, 15]. Therefore, we tested the hypothesis that the pattern of plasma miRNA expression would also allow the diagnosis and prediction of cardiac disease in MD patients. In a first step, we searched for those miRNAs that were known to be associated with either cardiovascular diseases and/or DMD/BMD based on the available literature data [30–38]. Compared to our control group, we identified seven significantly up-regulated plasma miRNAs (miR-206, miR-20b, miR-222, miR-26a, miR-342, miR-378a-3p, miR-378a-5p) and three down-regulated miRNAs (miR-221, miR-29a and miR-29c) in MD patients (Table 3). Since we did not detect any significant differences in miRNA expression in MD patients with mild myopathy (clinical degree 0-1 as defined in our Methods section) compared to those with advanced/severe myopathy (clinical degree of 2-3), the aforementioned seven up-regulated and three down-regulated miRNAs do not seem to reflect the skeletal myopathy status of our MD patients.

In the next step, we looked at those MD patients who had any abnormal CMR findings compared to those with completely normal CMR results. Interestingly, there were no significant differences in any of the measured plasma miRNAs (Table 6). Then, we focused first on “functional” cardiac parameters and thereafter on “structural” ones. Again, we did not detect significant differences in any of the measured plasma miRNAs in MD patients when those with an impaired systolic function were compared to those without (Table 9). However, patients with proof of myocardial scarring (LGE-positive) compared to those without (LGE-negative) showed a significant up-regulation of three miRNAs (miR-222, miR-26a and miR-378a-5p, Table 12). Importantly, there was no significant difference in the degree of skeletal myopathy or serum CK in LGE-positive MD patients compared to LGE-negative ones. Hence, the elevation of these three miRNAs in LGE-positive MD patients was not (only) caused by skeletal myopathy, but rather reflected cardiac disease.

Additional univariable analyses revealed a significant and substantial association between the expression level of two miRNAs (miR-222 and miR-26a) and the presence of myocardial scarring. Furthermore, multivariable regression analysis revealed three independent predictors for the presence of LGE – one being a miRNA (miR-222) in addition to a clinical (LV-EF) and a laboratory marker (elevated hs-Trop). Taken together, an up-regulation of three circulating miRNAs miR-222, miR-26a and miR-378a-5p is a useful signal for the presence of cardiac (structural) disease in MD patients – with miR-222 being the strongest and most important biomarker.

### Diagnostic value of plasma miRNAs compared to hs-Trop

In the present study, serum hs-Trop was a significant predictor of myocardial scarring – similar to miR-222. For further evaluation of the diagnostic value of the detected miRNAs in comparison to hs-Trop, we performed ROC analyses regarding the identification of LGE-positive MD patients. Area under the curve (AUC) values for the individual miRNAs (miR-222, miR-26a and miR-378a-5p) were close to 0.70. However, combining these three miRNAs resulted in an improved AUC value of 0.74 – compared to 0.75 in case of hs-Trop. However, the respective sensitivity, specificity and overall accuracy was 70, 73 and 70 % for the combination of these three miRNAs compared to 63, 87 and 65 % in case of hs-Trop. Hence, a combined approach based on the measurement of the three plasma miRNAs miR-222, miR-26a and miR-378a-5p seems to be at least as sensitive and accurate for the non-invasive diagnosis of cardiac disease in MD patients as the conventional hs-Trop measurement. Moreover, hs-Trop elevations may also have extra-cardiac causes (e.g. renal failure or myositis) – which so far were not described for the three identified circulating miRNAs. In future studies, the potential superior diagnostic as well as prognostic value of a miRNA-based approach for diagnosis of cardiomyopathy compared to established measurements (e.g. hs-Trop) needs to be evaluated in larger study groups.

### Comparison of present miRNA results to previous literature data

In the last years, several studies have been published addressing both pre-clinical animal models and humans as well as different biological materials such as skeletal muscle, heart muscle and serum/plasma. Previously published studies that (amongst others) addressed the three miRNAs miR-222, miR-26a and miR-378a-5p are summarized in the Additional file 3: Table S3. For example, miRNA analyses in the skeletal muscle of mdx mice and DMD patients revealed an up-regulation of miRNAs associated with muscle regeneration (e.g. miR-206) and inflammation (e.g. miR-222) and a down-regulation of miRNAs associated with muscle degeneration (e.g. miR-29c) [31].

Recently, Jeanson-Leh et al. analysed circulating miRNA profiles in golden retriever muscular dystrophy dogs and also evaluated the association of miRNA to cardiac disease [33]. Importantly, evaluation of cardiac disease was based only on functional analyses using echocardiography. Although a dysregulation of numerous miRNAs (such as miR-1, miR-95, miR-133, miR-208a/b, miR-206, miR-378 and miR-499) was detected in the serum of these dogs, there was no correlation between any miRNAs and cardiac functional parameters in this model. In agreement with this study, we also detected a dysregulation of some circulating miRNAs (e.g. miR-206 and miR-378) in the plasma of MD patients. Notably, we elucidated only a significant association between miRNA and cardiac involvement in MD patients when we looked specifically at structural – but not functional - abnormalities using myocardial scarring based on LGE-CMR. Obviously, miRNA profiling may improve the understanding of the pathophysiology of a certain disease if in depth phenotypic characterisation is considered.

### Limitations

Since the number of study patients was limited, replication of our data in an independent cohort is mandatory to demonstrate clinical utility. Moreover, in the present study we focused on selected miRNAs with evidence from previous studies. Thus, it cannot be excluded that additional miRNAs might even improve the predictive ability of the identified miRNA signature e.g. by comprehensive microarray profiling for miRNA expression. Finally, this was a pilot study and the results need verification in a larger cohort, with a multi-center approach and with consideration of clinical outcomes to define the respective role of miRNAs.

## Conclusions

Up-regulation of circulating miRNAs miR-222, miR-26a and miR-378a-5p indicates the presence of myocardial scars in MD patients. Plasma miR-222 appears to be a promising novel biomarker reflecting structural – but not functional – cardiac alterations in MD patients.

## Additional files

Additional file 1: Table S1. Primer information. (DOC 67 kb) Additional file 2: Table S2. Plasma miRNA results in MD patients according to the severity of skeletal myopathy. (DOC 40 kb) Additional file 3: Table S3. Summary of published data regarding miR-26a, miR-222 and miR-378-a5p. (DOC 126 kb)

## Abbreviations

BMD: Becker muscular dystrophy
CK: creatine kinase
CMR: cardiovascular magnetic resonance
DMD: Duchenne muscular dystrophy
hs-Trop: high-sensitive troponin
LGE: late-gadolinium-enhancement
LV: left ventricle
LV-EDV: left ventricular end-diastolic volume
LV-EF: left ventricular ejection fraction
LV-ESV: left ventricular end-systolic volume
MD: muscular dystrophy
NT-proBNP: brain natriuretic peptide
RV: right ventricle
RV-EDV: right ventricular end-diastolic volume
SSFP: steady-state-free-precession
