# Supplementary material for: Identification of cardiomyopathy associated circulating miRNA biomarkers in patients with muscular dystrophy using a complementary cardiovascular magnetic resonance and plasma profiling approach
Source: J Cardiovasc Magn Reson. 2016 May 6;18:25. doi: 10.1186/s12968-016-0244-3 (PMC4858897; doi:10.1186/s12968-016-0244-3)
Supplement: Additional file 1: Table S1. — Primer information. (DOC 67 kb) [file 12968_2016_244_MOESM1_ESM.doc]

**Supplemental Table 1: P**rimer information

| **miRBase ID** | **TaqMan miRNA Assay ID (Life technologies, USA)** | **TaqMan Assay name (Life technologies, USA)** |
| --- | --- | --- |
| hsa-miR-206 | 000510 | hsa-miR-206 |
| hsa-miR-133a-3p | 002246 | hsa-miR-133a |
| hsa-miR-208a-3p | 000511 | hsa-miR-208a |
| hsa-miR-208b-3p | 002290 | hsa-miR-208b |
| hsa-miR-31-5p | 002279 | hsa-miR-31 |
| hsa-miR-95-3p | 000433 | hsa-miR-95 |
| hsa-miR-539-5p | 001286 | hsa-miR-539 |
| hsa-miR-378a-5p | 000567 | hsa-miR-378a |
| hsa-miR-378a-3p | 002243 | hsa-miR-378a |
| hsa-miR-29c-3p | 000587 | hsa-miR-29c |
| hsa-miR-34c-5p | 000428 | hsa-miR-34c |
| hsa-miR-146b-5p | 001097 | hsa-miR-146b |
| hsa-miR-21-5p | 000397 | hsa-miR-21 |
| hsa-miR-16-5p | 000391 | hsa-miR-16 |
| hsa-miR-1-3p | 002222 | hsa-miR-1 |
| hsa-miR-499a-3p | 002427 | hsa-miR-499a-3p |
| hsa-miR-499a-5p | 001352 | hsa-miR-499a-5p |
| hsa-miR-451a | 001141 | hsa-miR-451 |
| hsa-miR-144* | 002148 | hsa-miR-144-5p |
| hsa-miR-26a-5p | 000405 | hsa-miR-26a |
| hsa-miR-29a-3p | 002112 | hsa-miR-29a |
| hsa-miR-15b-5p | 000390 | hsa-miR-15b |
| hsa-miR-195-5p | 000494 | hsa-miR-195 |
| hsa-miR-93-5p | 001090 | mmu-miR-93 |
| hsa-miR-221-3p | 000524 | hsa-miR-221 |
| hsa-miR-342-3p | 002260 | hsa-miR-342 |
| hsa-miR-20b-5p | 001014 | hsa-miR-20b |
| hsa-miR-222-3p | 002276 | hsa-miR-222 |

miRNA selection for quantification was based on literature data below:

Cacchiarelli, D., Legnini, I., Martone, J., Cazzella, V., Amico, A. D’, Bertini, E., and Bozzoni, I. (2011). miRNAs as serum biomarkers for Duchenne muscular dystrophy. EMBO Mol. Med. *3*, 258–265.

Corsten, M.F., Dennert, R., Jochems, S., Kuznetsova, T., Devaux, Y., Hofstra, L., Wagner, D.R., Staessen, J.A., Heymans, S., and Schroen, B. (2010). Circulating MicroRNA-208b and MicroRNA-499 reflect myocardial damage in cardiovascular disease. Circ. Cardiovasc. Genet. *3*, 499–506.

Devaux, Y., Mueller, M., Haaf, P., Goretti, E., Twerenbold, R., Zangrando, J., Vausort, M., Reichlin, T., Wildi, K., Moehring, B., et al. (2015). Diagnostic and prognostic value of circulating microRNAs in patients with acute chest pain. J. Intern. Med. *277*, 260–271.

Dickinson, B.A., Semus, H.M., Montgomery, R.L., Stack, C., Latimer, P.A., Lewton, S.M., Lynch, J.M., Hullinger, T.G., Seto, A.G., and van Rooij, E. (2013). Plasma microRNAs serve as biomarkers of therapeutic efficacy and disease progression in hypertension-induced heart failure. Eur. J. Heart Fail. *15*, 650–659.

Eisenberg, I., Eran, A., Nishino, I., Moggio, M., Lamperti, C., Amato, A.A., Lidov, H.G., Kang, P.B., North, K.N., Mitrani-Rosenbaum, S., et al. (2007). Distinctive patterns of microRNA expression in primary muscular disorders. Proc. Natl. Acad. Sci. U. S. A. *104*, 17016–17021.

Ellis, K.L., Cameron, V.A., Troughton, R.W., Frampton, C.M., Ellmers, L.J., and Richards, A.M. (2013). Circulating microRNAs as candidate markers to distinguish heart failure in breathless patients. Eur. J. Heart Fail. *15*, 1138–1147.

Endo, K., Weng, H., Naito, Y., Sasaoka, T., Takahashi, A., Fukushima, Y., and Iwai, N. (2013). Classification of various muscular tissues using miRNA profiling. Biomed. Res. Tokyo Jpn. *34*, 289–299.

Feng, H.J., Ouyang, W., Liu, J.H., Sun, Y.G., Hu, R., Huang, L.H., Xian, J.L., Jing, C.F., and Zhou, M.J. (2014). Global microRNA profiles and signaling pathways in the development of cardiac hypertrophy. Braz. J. Med. Biol. Res. Rev. Bras. Pesqui. Médicas E Biológicas Soc. Bras. Biofísica Al *47*, 361–368.

Gidlöf, O., Smith, J.G., Miyazu, K., Gilje, P., Spencer, A., Blomquist, S., and Erlinge, D. (2013). Circulating cardio-enriched microRNAs are associated with long-term prognosis following myocardial infarction. BMC Cardiovasc. Disord. *13*, 12.

Greco, S., De Simone, M., Colussi, C., Zaccagnini, G., Fasanaro, P., Pescatori, M., Cardani, R., Perbellini, R., Isaia, E., Sale, P., et al. (2009). Common micro-RNA signature in skeletal muscle damage and regeneration induced by Duchenne muscular dystrophy and acute ischemia. FASEB J. Off. Publ. Fed. Am. Soc. Exp. Biol. *23*, 3335–3346.

Greco, S., Perfetti, A., Fasanaro, P., Cardani, R., Capogrossi, M.C., Meola, G., and Martelli, F. (2012). Deregulated microRNAs in myotonic dystrophy type 2. PloS One *7*, e39732.

Hsu, A., Chen, S.-J., Chang, Y.-S., Chen, H.-C., and Chu, P.-H. (2014). Systemic approach to identify serum microRNAs as potential biomarkers for acute myocardial infarction. BioMed Res. Int. *2014*, 418628.

Hu, J., Kong, M., Ye, Y., Hong, S., Cheng, L., and Jiang, L. (2014). Serum miR-206 and other muscle-specific microRNAs as non-invasive biomarkers for Duchenne muscular dystrophy. J. Neurochem. *129*, 877–883.

Icli, B., Wara, A.K.M., Moslehi, J., Sun, X., Plovie, E., Cahill, M., Marchini, J.F., Schissler, A., Padera, R.F., Shi, J., et al. (2013). MicroRNA-26a regulates pathological and physiological angiogenesis by targeting BMP/SMAD1 signaling. Circ. Res. *113*, 1231–1241.

Ikeda, S., Kong, S.W., Lu, J., Bisping, E., Zhang, H., Allen, P.D., Golub, T.R., Pieske, B., and Pu, W.T. (2007). Altered microRNA expression in human heart disease. Physiol. Genomics *31*, 367–373.

Jaguszewski, M., Osipova, J., Ghadri, J.-R., Napp, L.C., Widera, C., Franke, J., Fijalkowski, M., Nowak, R., Fijalkowska, M., Volkmann, I., et al. (2014). A signature of circulating microRNAs differentiates takotsubo cardiomyopathy from acute myocardial infarction. Eur. Heart J. *35*, 999–1006.

Jeanson-Leh, L., Lameth, J., Krimi, S., Buisset, J., Amor, F., Le Guiner, C., Barthélémy, I., Servais, L., Blot, S., Voit, T., et al. (2014). Serum Profiling Identifies Novel Muscle miRNA and Cardiomyopathy-Related miRNA Biomarkers in Golden Retriever Muscular Dystrophy Dogs and Duchenne Muscular Dystrophy Patients. Am. J. Pathol. *184*, 2885–2898.

Li, C., Chen, X., Huang, J., Sun, Q., and Wang, L. (2015). Clinical impact of circulating miR-26a, miR-191, and miR-208b in plasma of patients with acute myocardial infarction. Eur. J. Med. Res. *20*, 58.

Li, X., Li, Y., Zhao, L., Zhang, D., Yao, X., Zhang, H., Wang, Y.-C., Wang, X.-Y., Xia, H., Yan, J., et al. (2014). Circulating Muscle-specific miRNAs in Duchenne Muscular Dystrophy Patients. Mol. Ther. Nucleic Acids *3*, e177.

Liu, X., Xiao, J., Zhu, H., Wei, X., Platt, C., Damilano, F., Xiao, C., Bezzerides, V., Boström, P., Che, L., et al. (2015). miR-222 is necessary for exercise-induced cardiac growth and protects against pathological cardiac remodeling. Cell Metab. *21*, 584–595.

Matkovich, S.J., Van Booven, D.J., Youker, K.A., Torre-Amione, G., Diwan, A., Eschenbacher, W.H., Dorn, L.E., Watson, M.A., Margulies, K.B., and Dorn, G.W. (2009). Reciprocal regulation of myocardial microRNAs and messenger RNA in human cardiomyopathy and reversal of the microRNA signature by biomechanical support. Circulation *119*, 1263–1271.

Mizuno, H., Nakamura, A., Aoki, Y., Ito, N., Kishi, S., Yamamoto, K., Sekiguchi, M., Takeda, S., and Hashido, K. (2011). Identification of muscle-specific microRNAs in serum of muscular dystrophy animal models: promising novel blood-based markers for muscular dystrophy. PloS One *6*, e18388.

Nagalingam, R.S., Sundaresan, N.R., Noor, M., Gupta, M.P., Solaro, R.J., and Gupta, M. (2014). Deficiency of cardiomyocyte-specific microRNA-378 contributes to the development of cardiac fibrosis involving a transforming growth factor β (TGFβ1)-dependent paracrine mechanism. J. Biol. Chem. *289*, 27199–27214.

Ramani, R., Vela, D., Segura, A., McNamara, D., Lemster, B., Samarendra, V., Kormos, R., Toyoda, Y., Bermudez, C., Frazier, O.H., et al. (2011). A micro-ribonucleic acid signature associated with recovery from assist device support in 2 groups of patients with severe heart failure. J. Am. Coll. Cardiol. *58*, 2270–2278.

Roberts, T.C., Blomberg, K.E.M., McClorey, G., Andaloussi, S. El, Godfrey, C., Betts, C., Coursindel, T., Gait, M.J., Smith, C.I.E., and Wood, M.J.A. (2012). Expression analysis in multiple muscle groups and serum reveals complexity in the microRNA transcriptome of the mdx mouse with implications for therapy. Mol. Ther. Nucleic Acids *1*, e39.

Roncarati, R., Viviani Anselmi, C., Losi, M.A., Papa, L., Cavarretta, E., Da Costa Martins, P., Contaldi, C., Saccani Jotti, G., Franzone, A., Galastri, L., et al. (2014). Circulating miR-29a, among other up-regulated microRNAs, is the only biomarker for both hypertrophy and fibrosis in patients with hypertrophic cardiomyopathy. J. Am. Coll. Cardiol. *63*, 920–927.

van Rooij, E., Sutherland, L.B., Liu, N., Williams, A.H., McAnally, J., Gerard, R.D., Richardson, J.A., and Olson, E.N. (2006). A signature pattern of stress-responsive microRNAs that can evoke cardiac hypertrophy and heart failure. Proc. Natl. Acad. Sci. U. S. A. *103*, 18255–18260.

van Rooij, E., Sutherland, L.B., Thatcher, J.E., DiMaio, J.M., Naseem, R.H., Marshall, W.S., Hill, J.A., and Olson, E.N. (2008). Dysregulation of microRNAs after myocardial infarction reveals a role of miR-29 in cardiac fibrosis. Proc. Natl. Acad. Sci. U. S. A. *105*, 13027–13032.

Sayed, A.S.M., Xia, K., Yang, T.-L., and Peng, J. (2013). Circulating microRNAs: a potential role in diagnosis and prognosis of acute myocardial infarction. Dis. Markers *35*, 561–566.

Sucharov, C., Bristow, M.R., and Port, J.D. (2008). miRNA expression in the failing human heart: functional correlates. J. Mol. Cell. Cardiol. *45*, 185–192.

Suh, J.H., Choi, E., Cha, M.-J., Song, B.-W., Ham, O., Lee, S.-Y., Yoon, C., Lee, C.-Y., Park, J.-H., Lee, S.H., et al. (2012). Up-regulation of miR-26a promotes apoptosis of hypoxic rat neonatal cardiomyocytes by repressing GSK-3β protein expression. Biochem. Biophys. Res. Commun. *423*, 404–410.

Tijsen, A.J., van der Made, I., van den Hoogenhof, M.M., Wijnen, W.J., van Deel, E.D., de Groot, N.E., Alekseev, S., Fluiter, K., Schroen, B., Goumans, M.-J., et al. (2014). The microRNA-15 family inhibits the TGFβ-pathway in the heart. Cardiovasc. Res. *104*, 61–71.

Togliatto, G., Trombetta, A., Dentelli, P., Cotogni, P., Rosso, A., Tschöp, M.H., Granata, R., Ghigo, E., and Brizzi, M.F. (2013). Unacylated ghrelin promotes skeletal muscle regeneration following hindlimb ischemia via SOD-2-mediated miR-221/222 expression. J. Am. Heart Assoc. *2*, e000376.

Vignier, N., Amor, F., Fogel, P., Duvallet, A., Poupiot, J., Charrier, S., Arock, M., Montus, M., Nelson, I., Richard, I., et al. (2013). Distinctive serum miRNA profile in mouse models of striated muscular pathologies. PloS One *8*, e55281.

Wei, C., Kim, I.-K., Kumar, S., Jayasinghe, S., Hong, N., Castoldi, G., Catalucci, D., Jones, W.K., and Gupta, S. (2013). NF-κB mediated miR-26a regulation in cardiac fibrosis. J. Cell. Physiol. *228*, 1433–1442.

Yu, B., Gong, M., Wang, Y., Millard, R.W., Pasha, Z., Yang, Y., Ashraf, M., and Xu, M. (2013). Cardiomyocyte protection by GATA-4 gene engineered mesenchymal stem cells is partially mediated by translocation of miR-221 in microvesicles. PloS One *8*, e73304.

Zaharieva, I.T., Calissano, M., Scoto, M., Preston, M., Cirak, S., Feng, L., Collins, J., Kole, R., Guglieri, M., Straub, V., et al. (2013). Dystromirs as serum biomarkers for monitoring the disease severity in Duchenne muscular Dystrophy. PloS One *8*, e80263.

Zanotti, S., Gibertini, S., Curcio, M., Savadori, P., Pasanisi, B., Morandi, L., Cornelio, F., Mantegazza, R., and Mora, M. (2015). Opposing roles of miR-21 and miR-29 in the progression of fibrosis in Duchenne muscular dystrophy. Biochim. Biophys. Acta *1852*, 1451–1464.

Zhang, H., Yang, H., Zhang, C., Jing, Y., Wang, C., Liu, C., Zhang, R., Wang, J., Zhang, J., Zen, K., et al. (2015). Investigation of microRNA expression in human serum during the aging process. J. Gerontol. A. Biol. Sci. Med. Sci. *70*, 102–109.

Zhang, Z., Li, J., Liu, B., Luo, C., Dong, Q., Zhao, L., Zhong, Y., Chen, W., Chen, M., and Liu, S. (2013). MicroRNA-26 was decreased in rat cardiac hypertrophy model and may be a promising therapeutic target. J. Cardiovasc. Pharmacol. *62*, 312–319.
