# Supplementary material for: Identification of cardiomyopathy associated circulating miRNA biomarkers in patients with muscular dystrophy using a complementary cardiovascular magnetic resonance and plasma profiling approach
Source: J Cardiovasc Magn Reson. 2016 May 6;18:25. doi: 10.1186/s12968-016-0244-3 (PMC4858897; doi:10.1186/s12968-016-0244-3)
Supplement: Additional file 2: Table S2. — Plasma miRNA results in MD patients according to the severity of skeletal myopathy. (DOC 40 kb) [file 12968_2016_244_MOESM2_ESM.doc]

**Supplemental Table 2:**

**Plasma miRNA results in MD patients according to the severity of skeletal myopathy**

| **miRNA**  **serum levels* (/10³)** | **Mild myopathy**  **N=45** | **Severe myopathy**  **N=18** | **p value** |
| --- | --- | --- | --- |
| **-206** | 15.54(3.75-50.24) | 33.31(6.11-79.06) | 0.36 |
| **-144** | 2.17(0.00-167.42) | 56.03(0.00-156.02) | 0.57 |
| **-146b-5p** | 81.86(0.00-209.46) | 3.71(0.00-125.01) | 0.08 |
| **-15b** | 0.00(0.00-18.78) | 0.97(0.00-27.61) | 0.72 |
| **-195** | 9.01(0.48-15.03) | 9.29(1.88-28.01) | 0.67 |
| **-20b** | 53.99(28.06-87.20) | 67.18(19.19-83.06) | 0.72 |
| **-21-5p** | 11.57(0.00-104.60) | 39.83(0.00-132.42) | 0.49 |
| **-221** | 0.00(0.00-11.27) | 0.00(0.00-27.23) | 0.54 |
| **-222** | 1833.18(1056.54-4651.33) | 2170.87(879.47-6187.27) | 0.59 |
| **-26a** | 326.81(126.07-554.70) | 235.45(121.09-515.64) | 0.61 |
| **-29a** | 0.00(0.00-0.00) | 0.00(0.00-0.00) | 0.23 |
| **-29c** | 0.00(0.00-2.57) | 0.00(0.00-0.00) | 0.11 |
| **-342** | 2442.67(1226.85-3896.99) | 2189.52(1006.92-5035.62) | 0.93 |
| **-378a-3p** | 19.31(0.66-146.77) | 67.43(18.47-301.62) | 0.22 |
| **-378a-5p** | 25.76(7.59-66.35) | 35.53(16.60-191.88) | 0.34 |
| **-451** | 184.13(78.30-338.52) | 190.17(52.11-334.21) | 0.82 |
| **-93** | 26.74(0.00-69.92) | 27.17(.0-44.42) | 0.72 |
