# Supplementary material for: Identification of cardiomyopathy associated circulating miRNA biomarkers in patients with muscular dystrophy using a complementary cardiovascular magnetic resonance and plasma profiling approach
Source: J Cardiovasc Magn Reson. 2016 May 6;18:25. doi: 10.1186/s12968-016-0244-3 (PMC4858897; doi:10.1186/s12968-016-0244-3)
Supplement: Additional file 3: Table S3. — Summary of published data regarding miR-26a, miR-222 and miR-378-a5p. (DOC 126 kb) [file 12968_2016_244_MOESM3_ESM.doc]

**Supplemental Table 3**. Summary of published data regarding miR-26a, miR-222 and miR-378-a5p

|  | **Analysed tissue** | **miR-26a** | **miR-222** | **miR-378a-5p** |
| --- | --- | --- | --- | --- |
| **Pre-clinical animal data** | **Skeletal muscle** | No data | Up-regulated in DMD *(mouse)^1, 2^* | Down-regulated in DMD *(mouse)*^1, 2^ |
|  |  |  | Up-regulation associated with muscle regeneration effects (*mouse*)^3^ |  |
|  | **Heart muscle** | Up-regulation associated with anti-fibrotic effect (*rat*)^4^ | Up-regulated in DMD (*mouse*)^2^ | Down-regulated in DMD *(mouse)*^1, 2^ |
|  |  | Down-regulated in LV hypertrophy (*rat*)^5^ | Up-regulation associated with cardioprotective and anti-apoptotic effects (*rat*)^6^ |  |
|  |  | Up-regulation with pro-apoptotic effect (*rat*)^7^ | Up-regulated in exercise model with myocyte proliferation effect (*mouse*)^8^ |  |
|  |  | Up-regulated in acute MI (*mouse*)^9^ | Up-regulation with cardioprotective effect after ischemic injury (*mouse*)^8^ |  |
|  | **Blood/ serum** | No data | No data | Up-regulated in DMD (*mouse*)^10^ |
| **Clinical data from patients** | **Skeletal muscle** | Down-regulated (DMD)^1, 11^ | Up-regulated in DMD^1, 11^ | No data |
|  | **Heart muscle** | Up-regulated in acute MI^9^ | Down-regulated in DCM, ICM^12^ | Up-regulated in LV systolic dysfunction^13^ |
|  |  | Down-regulation related to post LVAD recovery^14^ | Down-regulated in ICM with systolic dysfunction^15^ |  |
|  | **Blood** | Up-regulated in Tako-tsubo^16^ | Up-regulated with aging^17^ | Up-regulated in DMD^10^ |
|  |  | Down-regulated in acute MI^18^ |  | Down-regulated in LV hypertrophy^19^ |
|  |  | Up-regulated in HCM^20^ |  |  |

DMD – Duchenne muscular dystrophy; DCM – dilated cardiomyopathy; ICM – ischemic cardiomyopathy; LV – left ventricle; MI – myocardial infarction; LVAD – left ventricular assist device; HCM – hypertrophic cardiomyopathy.

Reference List

(1) Greco S, De SM, Colussi C et al. Common micro-RNA signature in skeletal muscle damage and regeneration induced by Duchenne muscular dystrophy and acute ischemia. *FASEB J* 2009 October;23(10):3335-46.

(2) Roberts TC, Blomberg KE, McClorey G et al. Expression analysis in multiple muscle groups and serum reveals complexity in the microRNA transcriptome of the mdx mouse with implications for therapy. *Mol Ther Nucleic Acids* 2012;1:e39.

(3) Togliatto G, Trombetta A, Dentelli P et al. Unacylated ghrelin promotes skeletal muscle regeneration following hindlimb ischemia via SOD-2-mediated miR-221/222 expression. *J Am Heart Assoc* 2013;2(6):e000376.

(4) Wei C, Kim IK, Kumar S et al. NF-kappaB mediated miR-26a regulation in cardiac fibrosis. *J Cell Physiol* 2013 July;228(7):1433-42.

(5) Zhang ZH, Li J, Liu BR et al. MicroRNA-26 was decreased in rat cardiac hypertrophy model and may be a promising therapeutic target. *J Cardiovasc Pharmacol* 2013 September;62(3):312-9.

(6) Yu B, Gong M, Wang Y et al. Cardiomyocyte protection by GATA-4 gene engineered mesenchymal stem cells is partially mediated by translocation of miR-221 in microvesicles. *PLoS One* 2013;8(8):e73304.

(7) Suh JH, Choi E, Cha MJ et al. Up-regulation of miR-26a promotes apoptosis of hypoxic rat neonatal cardiomyocytes by repressing GSK-3beta protein expression. *Biochem Biophys Res Commun* 2012 June 29;423(2):404-10.

(8) Liu X, Xiao J, Zhu H et al. miR-222 is necessary for exercise-induced cardiac growth and protects against pathological cardiac remodeling. *Cell Metab* 2015 April 7;21(4):584-95.

(9) Icli B, Wara AK, Moslehi J et al. MicroRNA-26a regulates pathological and physiological angiogenesis by targeting BMP/SMAD1 signaling. *Circ Res* 2013 November 8;113(11):1231-41.

(10) Vignier N, Amor F, Fogel P et al. Distinctive serum miRNA profile in mouse models of striated muscular pathologies. *PLoS One* 2013;8(2):e55281.

(11) Eisenberg I, Eran A, Nishino I et al. Distinctive patterns of microRNA expression in primary muscular disorders. *Proc Natl Acad Sci U S A* 2007 October 23;104(43):17016-21.

(12) Ikeda S, Kong SW, Lu J et al. Altered microRNA expression in human heart disease. *Physiol Genomics* 2007 November 14;31(3):367-73.

(13) Matkovich SJ, Van Booven DJ, Youker KA et al. Reciprocal regulation of myocardial microRNAs and messenger RNA in human cardiomyopathy and reversal of the microRNA signature by biomechanical support. *Circulation* 2009 March 10;119(9):1263-71.

(14) Ramani R, Vela D, Segura A et al. A micro-ribonucleic acid signature associated with recovery from assist device support in 2 groups of patients with severe heart failure. *J Am Coll Cardiol* 2011 November 22;58(22):2270-8.

(15) Sucharov C, Bristow MR, Port JD. miRNA expression in the failing human heart: functional correlates. *J Mol Cell Cardiol* 2008 August;45(2):185-92.

(16) Jaguszewski M, Osipova J, Ghadri JR et al. A signature of circulating microRNAs differentiates takotsubo cardiomyopathy from acute myocardial infarction. *Eur Heart J* 2014 April;35(15):999-1006.

(17) Zhang H, Yang H, Zhang C et al. Investigation of microRNA expression in human serum during the aging process. *J Gerontol A Biol Sci Med Sci* 2015 January;70(1):102-9.

(18) Li C, Chen X, Huang J, Sun Q, Wang L. Clinical impact of circulating miR-26a, miR-191, and miR-208b in plasma of patients with acute myocardial infarction. *Eur J Med Res* 2015;20:58.

(19) Chen Z, Li C, Xu Y, Li Y, Yang H, Rao L. Circulating level of miR-378 predicts left ventricular hypertrophy in patients with aortic stenosis. *PLoS One* 2014;9(8):e105702.

(20) Roncarati R, Viviani AC, Losi MA et al. Circulating miR-29a, among other up-regulated microRNAs, is the only biomarker for both hypertrophy and fibrosis in patients with hypertrophic cardiomyopathy. *J Am Coll Cardiol* 2014 March 11;63(9):920-7.
